# Limitations of learning in the proboscis reflex of the flower visiting syrphid fly *Eristalis tenax*

**DOI:** 10.1371/journal.pone.0194167

**Published:** 2018-03-20

**Authors:** Klaus Lunau, Lina An, Miriam Donda, Michele Hohmann, Leonie Sermon, Vanessa Stegmanns

**Affiliations:** 1 Institute of Sensory Ecology, Biology Department, Heinrich-Heine-University, Düsseldorf, Germany; 2 College of Plant Protection, Hebei Agricultural University, Baoding, China; University of Sussex, UNITED KINGDOM

## Abstract

Flower visiting *Eristalis* hoverflies feed on nectar and pollen and are known to rely on innate colour preferences. In addition to a preference for visiting yellow flowers, the flies possess an innate proboscis reflex elicited by chemical as well as yellow colour stimuli. In this study we show that the flies’ proboscis reflex is only triggered by yellow colour stimuli and not altered by conditioning to other colours. Neither in absolute nor in differential conditioning experiments the flies learned to associate other colours than yellow with reward. Even flies that experienced only blue nutrients during the first four days after hatching could not be trained to extend the proboscis towards other colours than yellow. The natural targets of the visually elicited proboscis reflex are yellow pollen and yellow anthers. One consequence of our findings is that flowers might advertise nectar and pollen rewards for *Eristalis* hoverflies by a yellow colour hue of nectar guides, nectaries, stamens or pollen. Alternatively, flowers might protect their pollen against *Eristalis* by displaying other pollen colours than yellow or direct flies by yellow pollen-mimicking floral guides towards nectar resources. Testing the proboscis extension of various hoverfly species in the field showed that only *Eristalis* hoverflies possess the proboscis reflex elicited by yellow colour hues.

## Introduction

Many nectar-feeding insect flower visitors such as bees [1], flies [2–5], moths [6] and butterflies [7–8] exhibit a proboscis extension reflex (PER) elicited by sugars such as glucose, fructose and sucrose. The PER is elicited by stimulation of taste receptors on the antennae, mouthparts, or tarsi of the legs with sugars [1–2, 7]. It is assumed that the PER helps flower visiting insects to find nectar sources. In laboratory studies it has been demonstrated that the PER can be conditioned to olfactory stimuli in bees [1] and butterflies [9]. Classical conditioning is based on an unconditioned stimulus eliciting the PER even in inexperienced animals and a conditioned stimulus presented shortly before, simultaneously, or shortly after the unconditioned stimulus that is learned via association in a number of training trials. In most studies odours are presented as conditioned stimuli in order to train the animals to respond by stimulation of the conditioned stimulus alone [1].

Visual stimuli are also known to elicit the proboscis reflex in flower visiting insects. Daumer [10] reported that honeybees extended the proboscis when passing a border between UV-reflecting and UV-absorbing areas of flowers. The extension of the proboscis to visual pollen stimuli is also shown by workers of the bumblebee *Bombus terrestris* when approaching flowers on the wing [11]. The innate proboscis reflex in the hoverfly *Eristalis tenax* is fine-tuned to yellow colour stimuli and inhibited by admixed blue or ultraviolet light [12]. In these studies about visual stimuli eliciting the proboscis reflex the researchers did not perform conditioning experiments.

The capability to learn to associate visual stimuli with sugar water reward in PER conditioning has only rarely been demonstrated. It is thought that harnessed insects are less capable to respond to visual stimuli. Niggebrügge et al. [13] found that honeybees discriminate colour stimuli if freely flying in operant conditioning settings much better as if restrained for classical conditioning experiments. The response to visual stimuli in the PER paradigm can be improved by cutting the antennae of harnessed bees [1]. However, Lichtenstein et al. [14] showed that classical PER conditioning to monochromatic light stimuli is also possible with intact bumblebee workers and drones using absolute and differential conditioning.

The hoverfly *Eristalis tenax* (Linnaeus 1758) (Syrphidae, Diptera) is known to extend the proboscis to yellow colours as well as to stimulation of the labellum and of the tarsi by sugar [12, 15]. In this study we performed classical conditioning experiments with the syrphid fly *E*. *tenax* using naïve flies reared from the pupae. In contrast to classical conditioning experiments with harnessed bees the proboscis reflex in *E*. *tenax* was tested in unharnessed individuals walking across an artificial flower which presented the visual colour stimuli. The flies were trained by exploiting the elicitation of the PER by sugar stimuli through presentation of sugar reward on top of the conditioned stimulus. In addition to the known response to sugars we tested the visually elicited proboscis reflex to pollen as suggested natural stimulus [12, 16–18]. We used different strength of training the visually elicited proboscis reflex in *Eristalis* including absolute conditioning, differential conditioning, and feeding freshly hatched flies exclusively with blue coloured nutrients, nectar and pollen. In addition we tested the eliciting of the proboscis reflex in hoverflies of several genera caught in the field immediately after capture and following starvation and training. Moreover we trained *E*. *tenax* flies to learn to discriminate artificial flowers only by means of the spot colour with the spot similar in size to the spots previously used for testing the proboscis extension reflex.

## Material and methods

### Keeping of the flies

**Fig 1:**
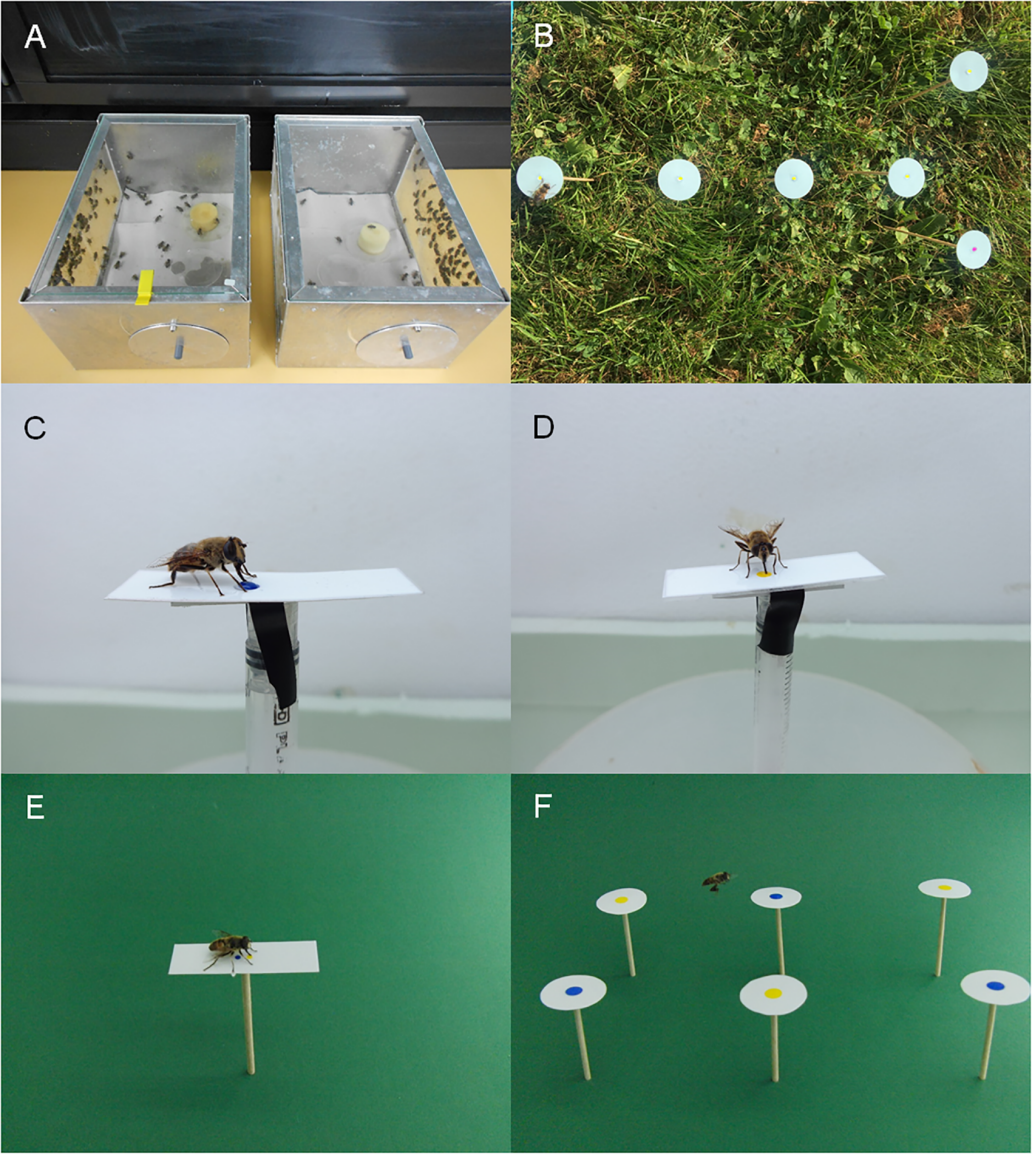
Fly keeping and testing. A) *Eristalis tenax* flies kept in cages. B) Outdoor setup for testing the landing response of trained flies to artificial flowers with different spot colour. C) Conditioning of the proboscis reflex in a fly to the blue spot colour. D) Conditioning of the proboscis reflex in a fly to the yellow spot colour. E) Dual choice test of the proboscis reflex in a trained fly. F) Indoor setup for testing the landing response of trained flies to artificial flowers with different spot colour of large-sized spots.

Larvae and pupae of *Eristalis tenax* were collected at a farm in Düsseldorf from mid of June to end of August in 2014, 2015 and 2017. The farm owner Karl-Peter Bergmeister gave permission to collect the flies on this site. The studies did not involve endangered or protected species. Only fully grown larvae were caught on their way to a dark place for pupation. Pupae were kept in metal boxes and the hatched imagoes were supplied with permanent access to water and diluted honey. Some larvae were transferred into a refrigerator and kept their up to 3 weeks at 8°C until they were released into the metal boxes for pupation and hatching of imagoes (Fig 1). All imagoes were kept naïve in regard to flowers and colours. Further treatments are explained in the description of the experiments.

The flies were either individually marked with honeybee queen tags (Opalithplättchen and shellac adhesive) or kept in separate metal boxes for identification. The tests were performed in the laboratory illuminated with flicker-free Osram Biolux T8 fluorescent tubes or in natural daylight.

The artificial flowers made of hp glossy photo paper (250g/m^2^) and printed with a Canon MX925 inkjet printer and original inks (S1 and S2 Figs and S1 Video). The experiments were conducted outdoors or in the laboratory.

### Training and testing of flies

For conditioning experiments all flies were trained and tested individually except for those flies that experienced blue coloured nutrients for 4 days. The flies that were tested with rectangular artificial flowers for proboscis extension were placed on the artificial flowers by means of a piece of filter paper. The flies that were tested with circular artificial flowers for landing response were placed on one artificial flower by means of a piece of filter paper and then landed on the next artificial flowers by themselves. Prior to that the piece of filter paper used for the placement of the flies was soaked in sugar water; by this means the flies were better motivated after placement with the filter paper. The flies were rewarded by droplets of sugar water placed on the small blue target spots. The droplets were smaller or about the same size of the spots. The concentration of the sugar water varied from 10% to 50% depending on the flies’ motivation. Keeping volume and concentration of sugar water rewards small ensured motivation of the flies to continue their search for sugar water during training procedures and tests. Moreover, the flies were not fed some hours before the trainings and tests in order to increase the motivation to search for food. Most flies extended the proboscis towards the reward offered on coloured spots after tapping with a front leg into the droplet of sugar water. A proboscis reaction was defined as the full extension of the proboscis with spread labella. If the flies did not take up the reward they were used for another trial later.

The rationale of the experiments was to demonstrate classical conditioning of the proboscis reflex in *E*. *tenax* flies to another colour hue than yellow. When one experiment had failed to demonstrate the conditioning the proboscis reflex, a further experiment was designed with more intense conditioning procedures implemented. When simple training was not successful, absolute and differential conditionings were tried, thereafter experience of blue nutrients for 4 days. The testing of field-caught flies was launched to test the response of other species than *E*. *tenax* and to test the conditioning with pollen instead of sugar water reward. The experiment with landing response of *E*. *tenax* was done to test the preference for the yellow spot colour in another context, the landing reaction.

The test arena was placed within a mosquito net. For acclimatization the flies were put into the mosquito net about half an hour before the training started. The artificial flowers were put into the test arena immediately before the training started. The training and test was carried out by a single experimenter who started training and testing with single flies by carrying them with a piece of filter paper to the artificial flower. Tested flies were removed from the mosquito net and not used for any further experiment. Most flies were cooperative during training and test; however, the flies were given several chances to conduct training and test. The time span between end of the training and beginning of the test was as short as necessary to replace the training setup by the test setup. Test setups were cleaned after each test and not used immediately thereafter again. Each experiment was developed by another experimenter. In the various experiments, changes in background colour, spot size and others were endorsed in order to minimize influence by the experimental conditions. The proportions and colour pattern of the artificial flowers and its spectral reflectance properties are shown as insets in the figures of the results chapter and in the supplement (S1 and S2 Figs).

### Dual choice of trained flies

Rectangular artificial flowers were 5.0 x 1.5cm in size and had two 2mm sized spots in the middle (S1 Fig). The artificial flowers were attached to a 9cm long wooden stick and presented against a green background made of cardboard in the laboratory. Thirty flies were trained three times by walking across the artificial flower with two blue spots each with a droplet of sugar water. It was ensured that the flies sucked the sugar water. Only very few flies that did not imbibe the sucrose solution were excluded from the analysis. As a control, 30 flies were trained three times by walking across the artificial flower with two yellow spots each with a droplet of sugar water. Immediately after training the flies were tested for their proboscis response. In the test no reward was offered on artificial flowers displaying one blue and one yellow spot. The flies were placed by means of a piece of filter paper soaked in sugar water to the end of the artificial flower. Each tested fly had to walk once from each end across the artificial flower, such that once the fly encountered the blue spot before the yellow spot and once the yellow spot before the blue spot. Each fly was tested only once. The proboscis extension towards one to the spots was scored.

In an alternative setup imagoes of *E*. *tenax* were trained to walk on a T-shaped light grey artificial flower (with 2 short arms of 2.5cm each and 1 long arm of 5.0cm) towards a rewarding colour spot (8mm) presented at the end of the short arms three times (S1 Fig). In the training both short arms displayed the same spot colour, blue or yellow, whereas in the test the two arms displayed different colours, blue and yellow. Thirty flies were trained to the yellow spot colour and 30 flies to the blue spot colour. The flies were placed by means of a piece of filter paper to the end of the long arm. The proboscis extension towards one to the spots was scored.

### Absolute and differential conditioning

This experiment focused on the conditioning of the flies’ proboscis reflex towards the blue colour. Rectangular artificial flowers made of photo paper were 5.0 x 1.6cm in size and had one 3mm sized spot in the middle. The artificial flowers were attached to a 9cm long wooden stick and presented against a white background made of cardboard. For absolute conditioning the flies walked 10 times across the artificial flower which offered a droplet of sugar water on the blue spot. It was ensured that the flies sucked the sugar water. Immediately after the training the flies were tested first on an artificial flower with a blue spot, then 5 min later again on an artificial flower with a blue spot, and then on an artificial flower with a yellow spot as a control (S1 and S2 Figs). The flies were trained and tested this way three days in a row. Twenty flies were tested following absolute conditioning.

For differential conditioning the flies walked 5 times across an artificial flower which offered a droplet of sugar water on the blue spot and alternately 5 times across an artificial flower which offered a droplet of quinine solution (0.02%) on the yellow spot. Quinine is known to be bitter-tasting for insects and was used here as a punishment. It was checked that the flies sucked the sugar water as a reward and that the flies did not drink the quinine solution after probing. Twenty flies were tested following differential conditioning. Immediately after the training the flies were tested first on an artificial flower with a blue spot, then 5 min later again on an artificial flower with a blue spot, and then on an artificial flower with a yellow spot as a control (S1 and S2 Figs). The flies were trained and tested this way two days in a row. The proboscis extension towards the spot was scored.

In the tests following absolute conditioning a water droplet was placed on the yellow or blue spot. In a control experiment the tests were done without a droplet placed on the yellow or blue spot in order to show how much the glistening liquid contributed to the elicitation of the proboscis reflex. In the tests following differential conditioning no water droplet was placed on the yellow or blue spot. All tests were conducted in the laboratory. The proboscis extension towards the spot was scored.

### Proboscis extension reflex in flies grown in a colour controlled environment

Newly hatched flies were fed for 4 days exclusively with blue sugar water and blue pollen. The sugar water was coloured with blue food colourant. The blue pollen was manually selected from commercial Spanish bee pollen collected by honeybees. Water was provided from wet paper towels placed in the cage. It was not controlled whether individual flies imbibed the sugar water and fed on the pollen, but according to our knowledge from fly-keeping the flies will not survive 4 days without taking any sugar water. In addition, the flies were given once a day a nutritious yellow solution of pollen and sugar water embittered with quinine (0.02%). The flies did not drink the embittered solution. In the fifth day the flies were treated in the laboratory as described for differential conditioning, but tested only immediately after the conditioning and 5 min later (S1 Fig).

### Proboscis extension reflex in experienced field-caught flies

Free and experienced hoverflies were caught in the field and the proboscis extension reflex was tested on rectangular white artificial flowers made of photo paper were 5.8 x 1.7cm, 5.8 x 1.0cm, or 5.8 x 0.7cm in size depending on the flies’ body size (S1 Fig). The rationale of this treatment was that the flies walking over the artificial flower should go across the coloured spots. The large artificial flower had 5 yellow spots 2.7mm in diameter, the middle one 5 yellow spots 2.0mm in diameter, and the small one 5 yellow spots 1.6mm in diameter. The artificial flowers were offered in a tunnel of UV-transmitting foil in order to avoid the escape of the flies. The flies were placed in a chamber for some time to calm down before being released to walk across the artificial flower. Each fly was tested 3 times, first immediately after being caught in the field, second after 2 hours of starvation in a cooled dark box and third after being fed with yellow sunflower pollen on a training artificial flower. The pollen was offered on a modified artificial flower in 0.5mm deep cavities instead of spots fabricated by stamping out the spots. The rationale of this treatment was to increase the motivation to feed. The cooling was necessary to ensure survival on hot days. The data of flies of different species of the same genus were pooled for evaluation if no significant differences in response between the species were found. The proboscis extension towards the spot was scored.

### Landing response to artificial flowers with different spot colour

Imagoes of *E*. *tenax* that hatched in the laboratory were trained four times to find sugar water on light yellow or light blue artificial flowers (3cm) displaying a central spot (2mm) of different colour. The four training artificial flowers were arranged in a row with 10cm space between each other so that the trained flies visited one after the other. The two test artificial flowers were placed 10cm apart from the last training artificial flower and 10cm apart from each other (S1 and S2 Figs). The only variable in the experiments was the colour of the spots. In one set of experiments the flies were rewarded on the deep yellow spot of the artificial flowers four times and then given the dual choice between an artificial flower with a deep yellow spot and an artificial flower with a white spot. In a reciprocal test the flies were rewarded on the white spot and then given the same dual choice. In another set of experiments the white spot colour was replaced by a violet spot colour. The landing reaction on the artificial flowers was scored.

In an alternative setup imagoes of *E*. *tenax* were trained four times to find sugar water on light grey artificial flowers (3cm) displaying a large central spot (8mm) of deviant colour. Starting from one rewarding artificial flower the flies were given three dual choices between artificial flowers displaying the trained spot colour and an alternative spot colour. This test was performed twice resulting in a maximum of 6 choices for each colour (S1 and S2 Figs and S1 Video). For the training trial the same arrangement of artificial flowers was used, but with only one spot colour. The distance between the artificial flowers was 10cm. Thirty flies were trained to the blue spot colour and 30 flies were trained to the yellow spot colour. The landing reaction on the artificial flowers was scored. The behaviour of the flies during the test is shown in the supplement.

## Results

### Dual choice of trained flies

**Fig 2:**
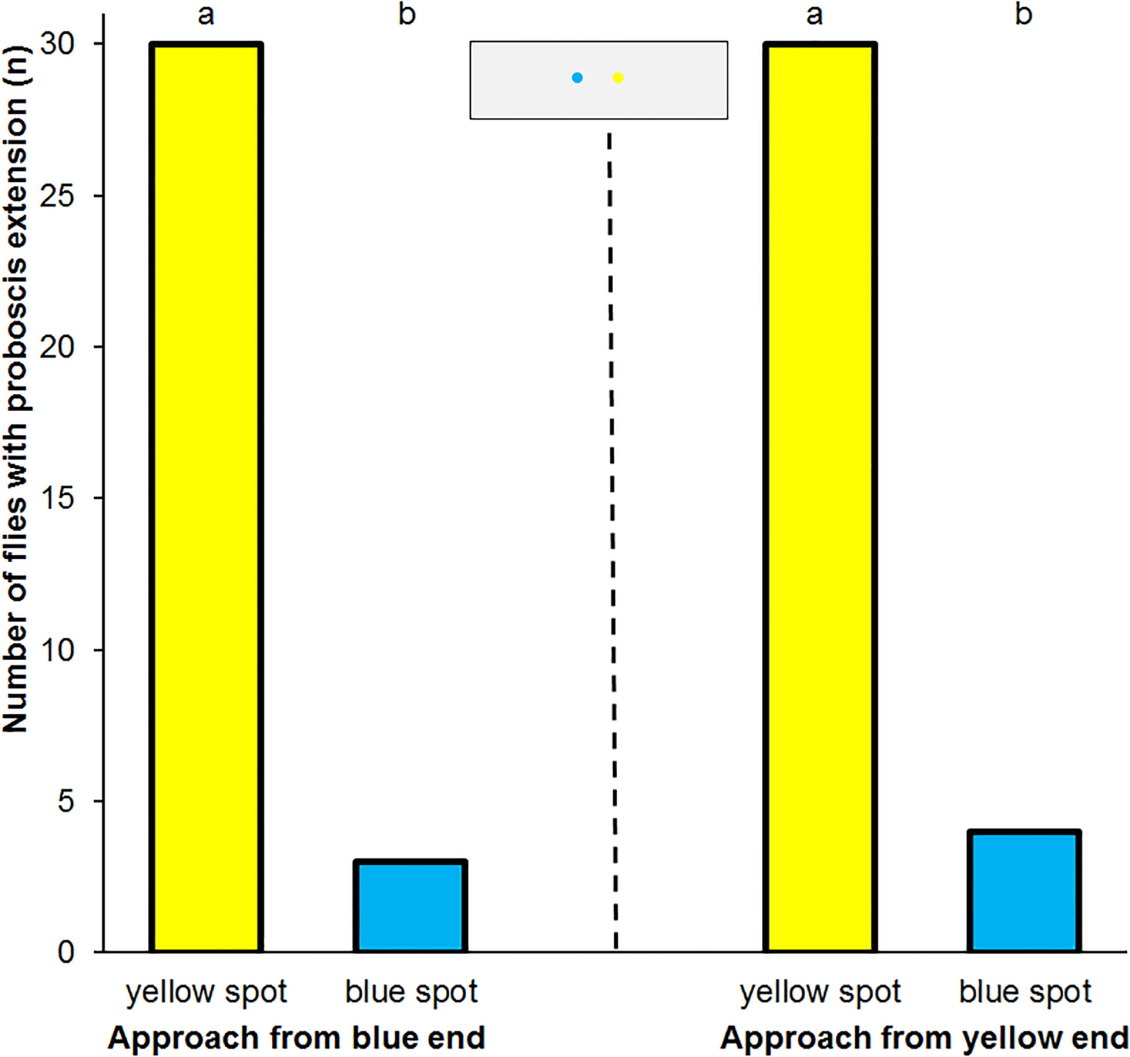
Proboscis reflex following training. Frequency of proboscis extension in a dual choice test with blue and yellow spots in *Eristalis tenax* that experienced absolute conditioning with reward on blue colours (n = 30). Each fly walked across the artificial flower once from each side. The control experiment revealed an identical result (n = 30). Different letters denote significant differences due to a two-tailed Fisher’s Exact test (p<0.001). The inset shows the artificial flower used in the tests.

All 30 flies trained to yellow extended the proboscis towards the yellow spot irrespective of the side of approach. When approaching the blue spot first 3 flies extended the proboscis to the blue spot; when approaching the yellow spot first 4 flies extended the proboscis in addition to the yellow also to the blue spot (Fig 2). The 30 flies trained to blue showed similar results: All flies extended the proboscis towards the yellow spot irrespective of the side of approach and only 3, resp. 4 flies extended the proboscis towards the blue spot. In each test 15 males and 15 females were tested. The frequency of proboscis extension did not differ between males and females in both tests (Chi-square test, p = 0.682, resp. p = 0.720).

**Fig 3:**
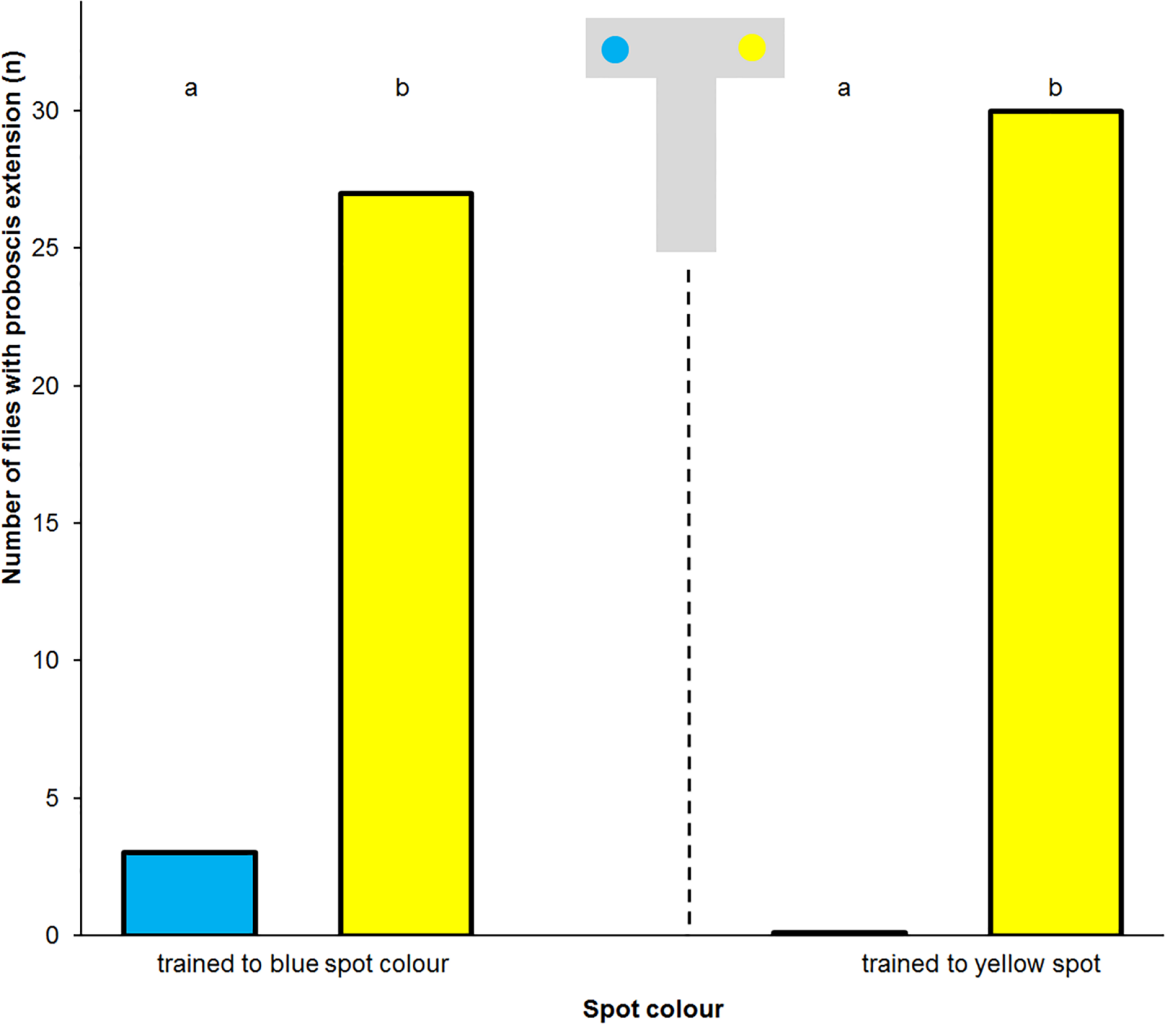
Walking direction following training. Frequency of *Eristalis tenax* flies walking towards a yellow or blue spot displayed on either arms of a T-shaped artificial flower dependent of training (n = 60). Different letters denote significant differences due to a two-tailed Chi-square test (p<0.01). The inset shows the artificial flower used in the tests.

All 30 flies trained to walk to the arm of the T-shaped artificial flower presenting reward on a yellow spot chose the arm with the yellow spot in the test (Fig 3; Chi-square test, p<0.0001). Among the 30 flies trained to walk to the arm of the T-shaped artificial flower presenting reward on a blue spot 3 flies chose the arm with the blue spot in the test, whereas 27 flies walked towards the yellow spot (Chi-square test, p = 0.0019).

### Absolute and differential conditioning

**Fig 4:**
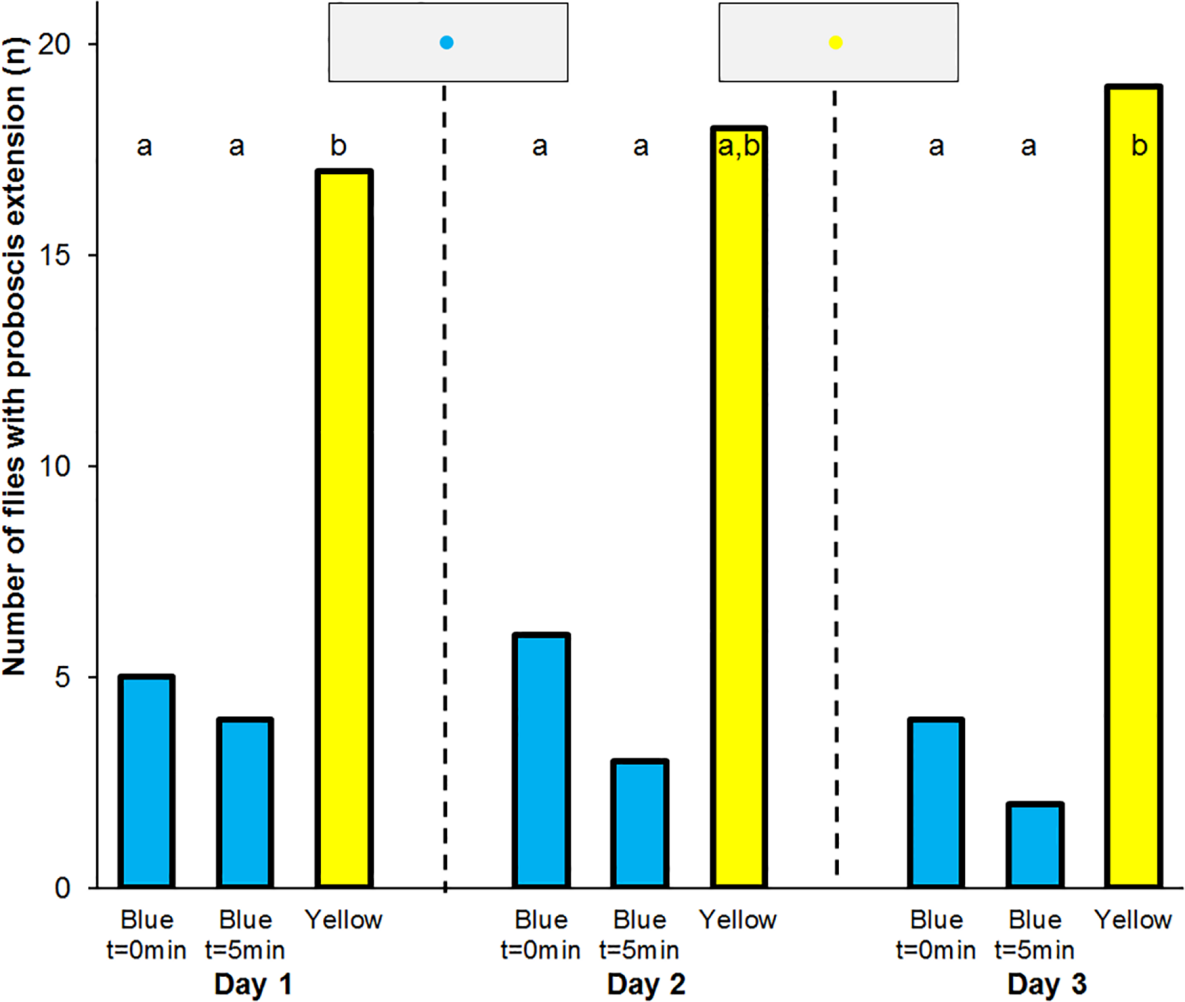
Absolute conditioning of the proboscis reflex to blue. Frequency of proboscis extension to blue and yellow spots in *Eristalis tenax* that experienced absolute conditioning with reward on blue colours (n = 20). Different letters denote significant differences due to a one-tailed Sign test (p<0.05). Blue t = 0min denotes the test to blue spots immediately after training, Blue t = 5min that 5 minutes later, Yellow the control test to yellow spots. The insets show the artificial flowers used in the tests.

Of the 20 flies that experienced absolute conditioning to blue 17 individuals extended their proboscis to the yellow spot, and 5 individuals extended their proboscis to the blue spot immediately after training. Only 4 flies responded to the blue spot 5 min later. Following additional absolute conditioning to blue the next and following day the flies responded similarly (Fig 4).

**Fig 5:**
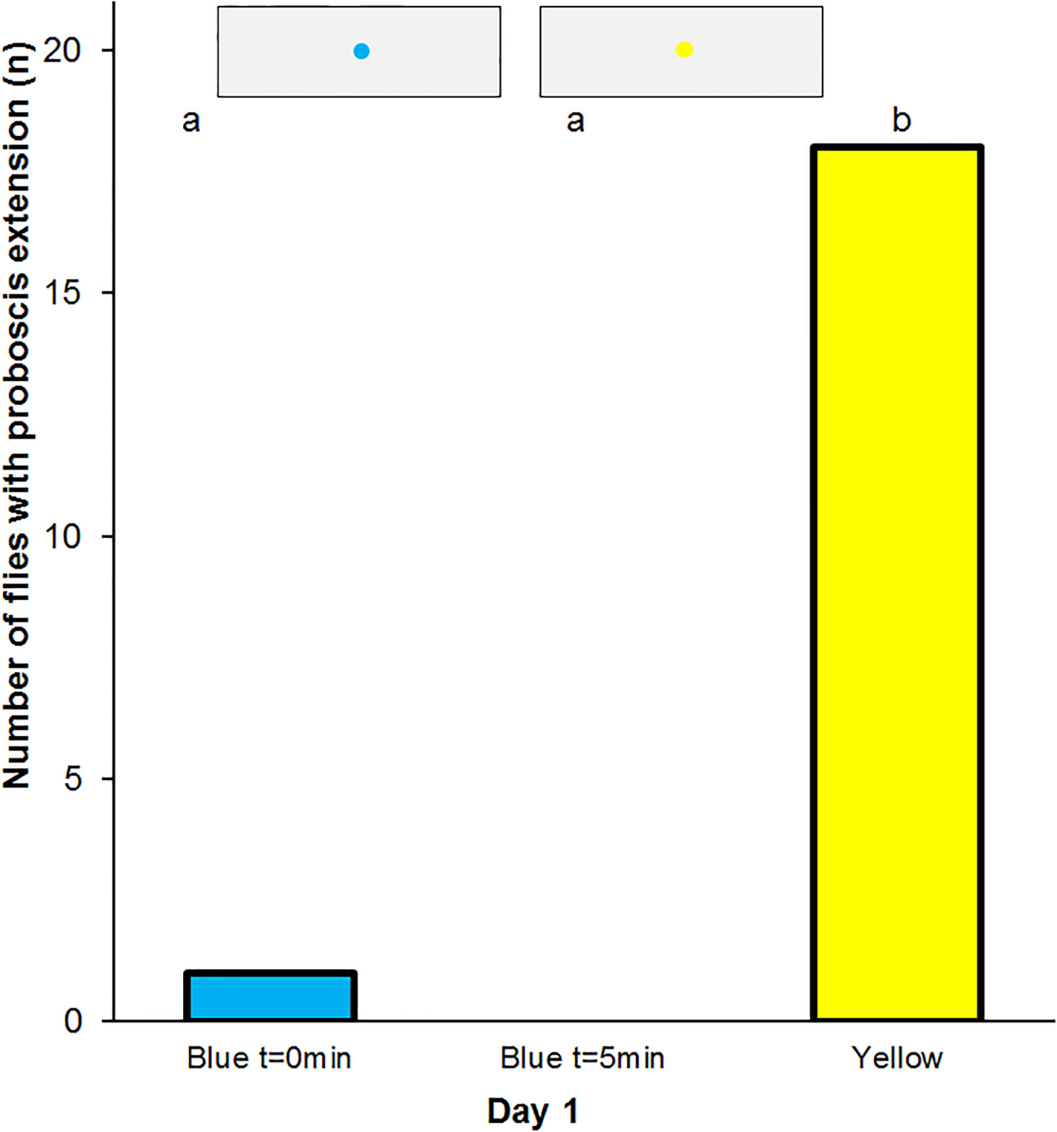
Absolute conditioning of the proboscis reflex to blue (control). Frequency of proboscis extension to blue and yellow spots in *Eristalis tenax* that experienced absolute conditioning with reward on blue colours and were tested on artificial flowers offering no droplet of a liquid (n = 20). Different letters denote significant differences due to a one-tailed Sign test (p<0.01). Blue t = 0min denotes the test to blue spots immediately after training, Blue t = 5min that 5 minutes later, Yellow the control test to yellow spots. The insets show the artificial flowers used in the tests.

In the control experiment 20 flies experienced absolute conditioning and were tested on artificial flowers with spots that did not provide a droplet of water on the spot. In the test 18 individuals extended their proboscis to the yellow spot, and 1 individual extended its proboscis to the blue spot immediately after training (Fig 5).

**Fig 6:**
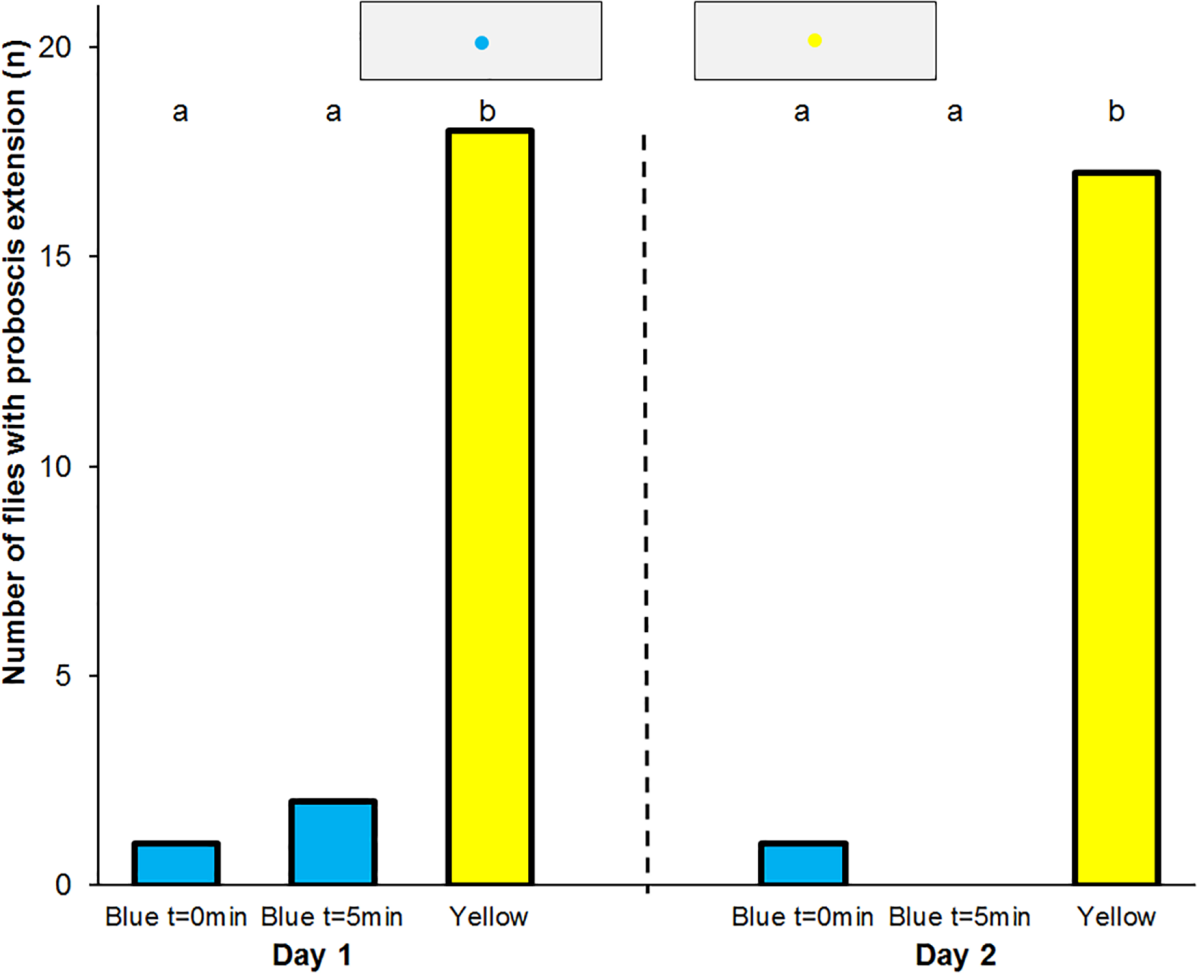
Differential conditioning of the proboscis reflex. Frequency of proboscis extension to blue and yellow spots in *Eristalis tenax* that experienced differential conditioning with reward on blue and punishment on yellow colours (n = 20). Different letters denote significant differences due to a one-tailed Sign test (p<0.01). Blue t = 0min denotes the test to blue spots immediately after training, Blue t = 5min that 5 minutes later, Yellow the control test to yellow spots. The insets show the artificial flowers used in the tests.

Of the 20 flies that experienced differential conditioning to blue 18 individuals extended their proboscis to the yellow spot, and 1 individual extended its proboscis to the blue spot immediately after training. Only 2 flies responded to the blue spot 5 min later. Following differential absolute conditioning to blue the next day the flies responded similarly (Fig 6).

### Proboscis extension reflex in flies grown up in a colour controlled environment

**Fig 7:**
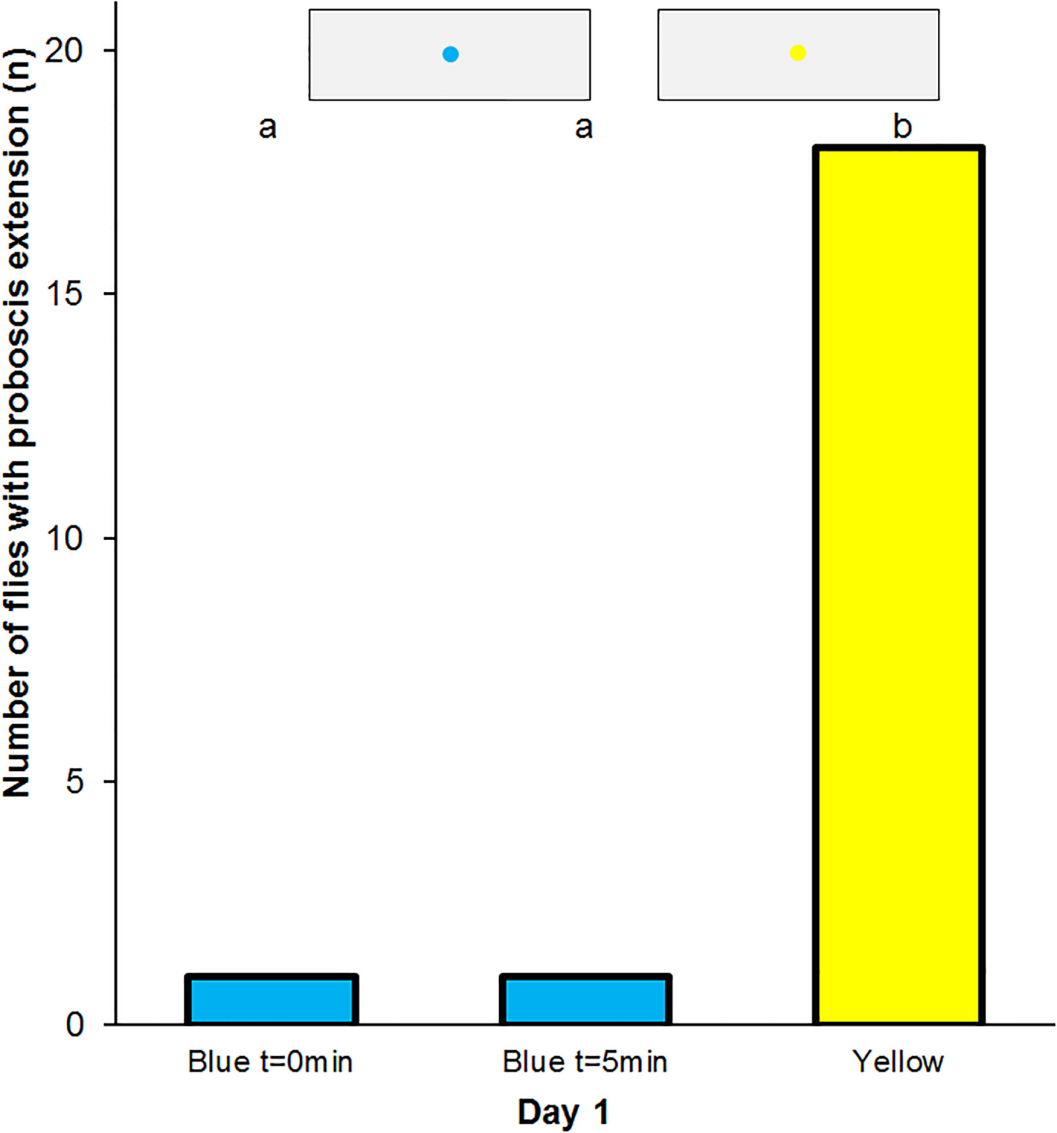
Extreme differential conditioning of the proboscis reflex. Frequency of proboscis extension to blue and yellow spots in *Eristalis tenax* fed with blue coloured nutrients and experienced differential conditioning with reward on blue and punishment on yellow colours (n = 20). Different letters denote significant differences due to a one-tailed Sign test (p<0.01). Blue t = 0min denotes the test to blue spots immediately after training, Blue t = 5min that 5 minutes later, Yellow the control test to yellow spots. The insets show the artificial flowers used in the tests.

Imagoes of *E*. *tenax* that were fed with blue pollen and blue sugar water for four days after hatching and then experienced a differential conditioning with reward on blue and punishment on yellow colours significantly extended their proboscis more often to yellow than to blue spots. Eighteen of 20 tested flies showed the proboscis reflex towards the yellow spot and only 1 fly responded to blue spot immediately after training and 5 min later (Fig 7).

### Proboscis extension reflex in experienced field-caught flies

**Fig 8:**
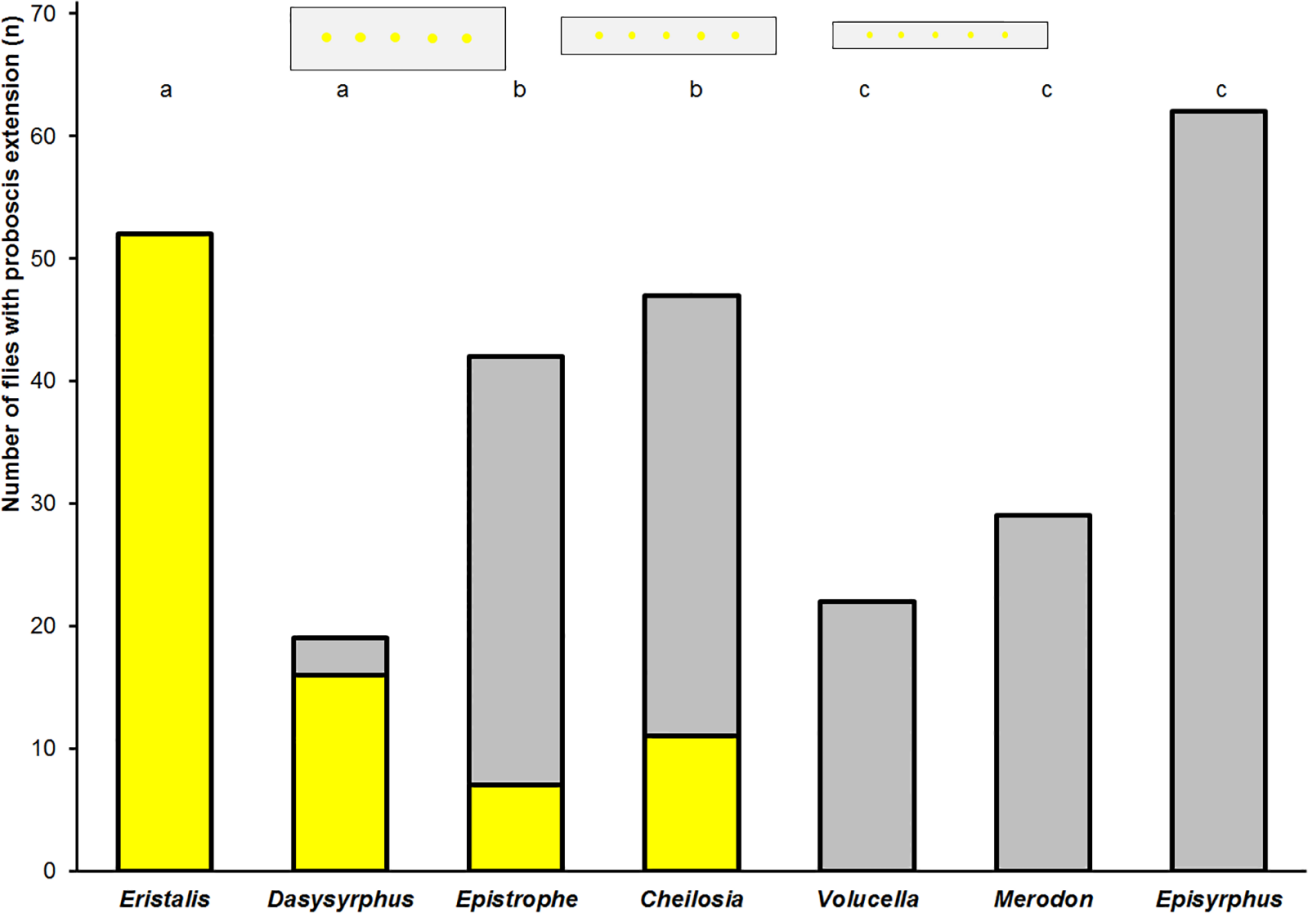
Proboscis reflex of field-caught hoverflies. Frequency of experienced hoverflies flies of various genera caught in the field that responded or did not respond with proboscis extension to the yellow spots following starvation for 2 hours. The yellow colour denotes flies that extended their proboscis towards at least one of the yellow spots, the grey colour denotes flies that did not. Different letters denote significant differences due to a two-tailed Fisher’s Exact test (p<0.01). The insets show the three artificial flowers used in the tests varying only in width and spot size.

Imagoes of hoverflies caught in the field tested were *Eristalis tenax* (n = 8), *Er*. *pertinax* (n = 44), *Epistrophe niticollis* (n = 18), *Ep*. *elegans* (n = 13), *Ep*. *grossulariae* (n = 11), *Dasysyrphus lunulatus* (n = 7), *D*. *venustus* (n = 12), *Cheilosia fasciata* (n = 47), *Volucella inflata* (n = 22), *Merodon equestris* (n = 29), and *Episyrphus balteatus* (n = 62). Imagoes belonging to the same genus were merged for evaluation. 98% of the *Eristalis* flies showed a proboscis reflex towards the yellow spots flies, 9% of *Dasysyrphus* flies, and 11% of *Cheilosia fasciata; individuals* of the genus *Epistrophe*, *Volucella inflata*, *Merodon equestris*, and *Episyrphus balteatus* did not respond when tested immediately after they were caught. After 2 hours of starvation 100% of the *Eristalis* flies, 84% of *Dasysyrphus* flies, 17% of *Epistrophe* flies and 23% of *Cheilosia fasciata* showed a proboscis reflex towards the yellow spots, whereas the other flies did not respond (Fig 8, S1 Fig). After feeding pollen only *Eristalis* flies showed the proboscis reflex towards the yellow spots.

### Landing response to artificial flowers with different spot colour

**Fig 9:**
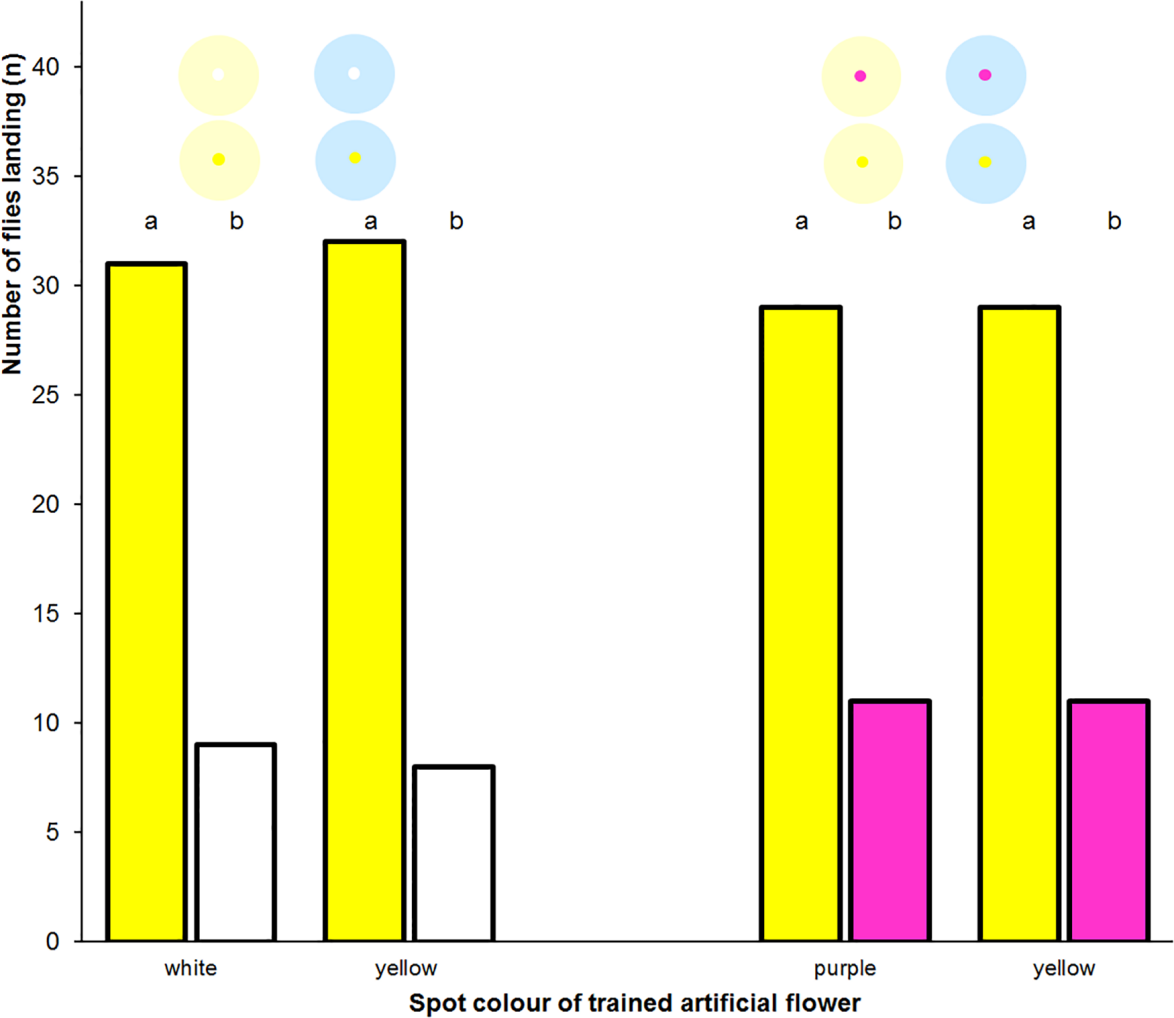
Landing response following training to small spots. Frequency of landing in trained *Eristalis tenax* flies that responded to a dual choice test with spot colour as a variable. Results for light blue and light yellow artificial flowers were pooled. Different letters denote significant differences due to a two-tailed Fisher’s Exact test (p<0.05). The insets show the artificial flowers used in the tests.

Imagoes of *E*. *tenax* that experienced four times of absolute conditioning of the landing reaction to an artificial flower and were rewarded on the coloured spot were given a dual choice immediately thereafter (S1 Fig). The flies preferred to land on the artificial flower displaying a yellow spot irrespective of the colour of the artificial flower and the training (Fig 9).

**Fig 10:**
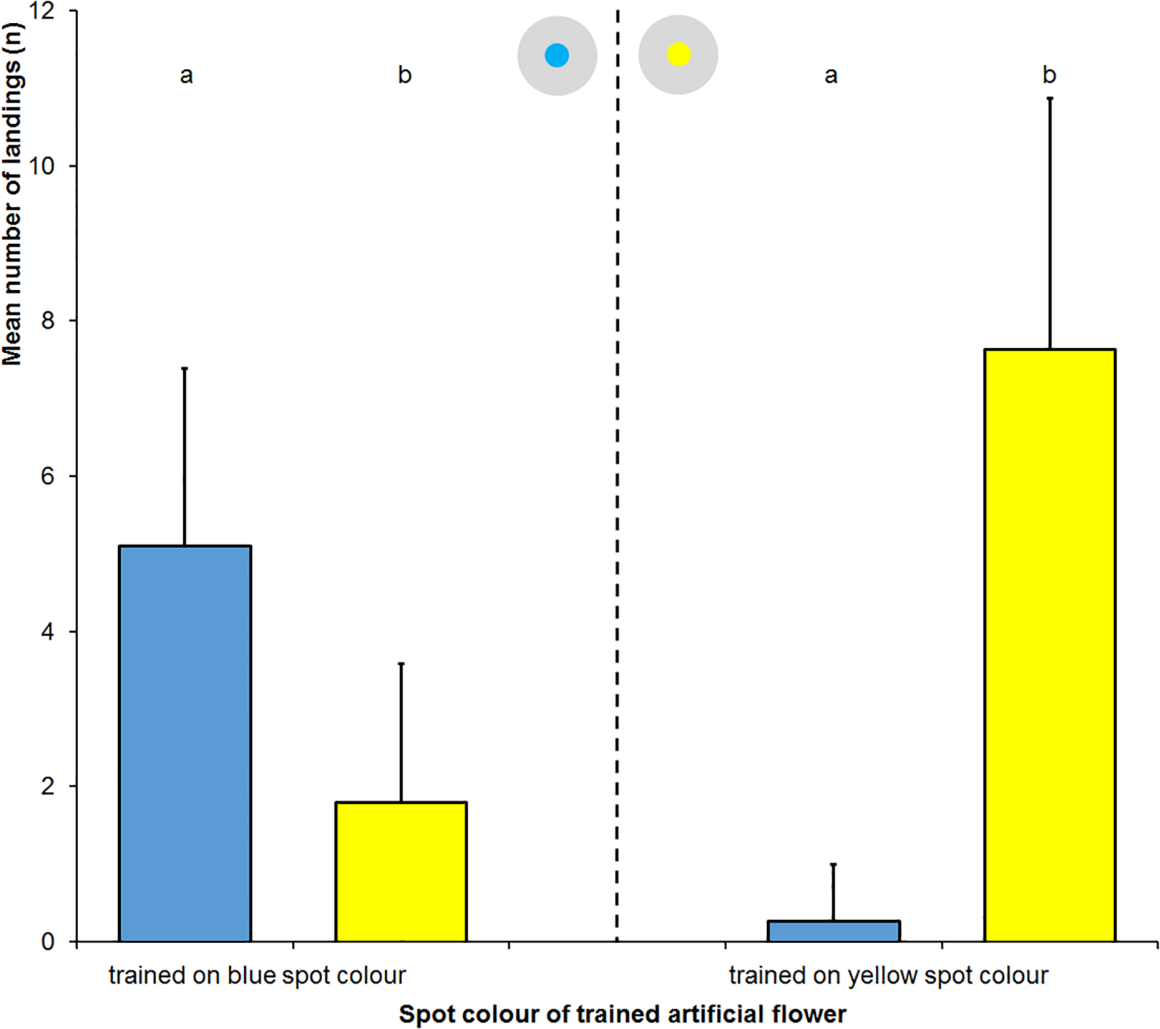
Landing response following training to large spots. Mean number of landings and positive standard deviation of *Eristalis tenax* trained to land on grey artificial flowers with large coloured spots dependent of training (n = 60). Different letters denote significant differences due to a two-tailed Mann Whitney test (p<0.0001). The inset show the artificial flowers used in the tests.

Using light grey artificial flowers with large coloured spots (S1 Fig) the flies trained to the blue spot colour landed on artificial flowers (S1 Video) with a blue spot on average 5.1±2.3 times and on artificial flowers with a yellow spot on average 1.8±1.8 times (p<0.0001, two-tailed Mann-Whitney test). The flies trained to the yellow spot colour landed on artificial flowers with a blue spot on average 0.3±0.7 times and on artificial flowers with a yellow spot on average 7.6±3.3 times (Fig 10); p<0.0001, two-tailed Mann-Whitney test).

## Discussion

Our results show that training, absolute conditioning and differential conditioning, repeated differential conditioning as well as exclusive nutrition with blue coloured pollen and sugar water do not alter the innate proboscis reflex towards yellow colours. Reports of a failure of conditioning the proboscis reflex in insects are rare, however, there are pitfalls in the protocol studying the proboscis extension response that might reduce the percentage of responding test animals [19–20]. Abramson et al. [21] described the failure of classical conditioning of the PER to olfactory conditioned stimuli for one-day-old Africanized honey bees and adult stingless bees (*Melipona scutellaris*). Vorel and Pitts-Singer [22] found no proboscis reflex to 25% sucrose solution in solitary megachilid bees, although they tested different methods to restrain the bees. Giurfa and Sandoz [1] described limitations of the proboscis extension response in honeybees and concluded that olfactory, mechanosensory and thermal stimuli have been more successfully tested as unconditioned stimuli as compared to visual stimuli.

The harnessing of the tested insects, i.e. the immobilization of the tested insects, is considered an important factor limiting the training to visual stimuli. While harnessed honeybees were not capable of learning a direct association between colour and sugar water reward, the bees learned to use colours to modulate olfactory conditioning of the proboscis reflex [23]. It has been reported that bees with cut antennae can be trained to learn visual cues, but even after ablation of the antennae, learning success in these PER experiments was limited [24–25]. By contrast, the stinger extension reflex in honeybees could successfully be conditioned to colour stimuli [26]. Jernigan et al. [27] could successfully use colours as conditioned stimulus in PER-experiments with Africanized bees, but only for distinct colour stimuli. Similar results hold for bumblebees (*Bombus impatiens*) [28]. Pretraining with visual colour stimuli did not modulate olfactory learning in tests with PER responses of harnessed honeybees [29]. In learning experiments with harnessed bees in which pollen was used as an unconditioned stimulus, honeybees did not learn to associate a neutral odour with pollen reward indicating that pollen has a proboscis extension releasing function, but does not reinforce olfactory learning in the context of the PER [30]. The tested animals in this study were freely walking *Eristalis* imagoes, which means that restrictions of its liberty of action should not play a role. Indeed, it has previously been demonstrated that *Eristalis* flies use yellow spots also to locate food sources and walk towards them [31–32]. This means that passing the border from a non-triggering colour to a triggering colour stimulus might be decisive for the elicitation of the proboscis response. This stimulus property cannot be applied to harnessed flies.

It is striking that that visual PER leads to more limited performance as compared to visual conditioning of freely flying bees. The performance of harnessed bees was much improved in the presence of the motion stimulus [33–34]. Colour learning experiments in freely flying bees [35] and experiments using a T-maze [36] show that bees can learn to associate colour stimuli with a reward. In this study trained flies preferred to walk towards yellow spots even if they had been trained to another spot colour, and to land on artificial flowers with a yellow spot even if they had been trained to artificial flowers with another spot colour. This result suggests that the yellow colour as a target for proboscis extension is already important for the approaching and landing flies. Indeed the yellow spot colour is known to attract *Eristalis* flies moving on artificial flowers [31]. By contrast, Lunau [37] has shown that the flies learn the presence or absence of spots for triggering landing behaviour. This is not in agreement with the finding that hoverflies can learn to respond to floral colour change of small-sized floral guides in *Myosotis sylvatica* [38]. However, our experiment in which 8mm-sized instead of 2mm-sized spots were used, demonstrated that *Eristalis* flies are able to use the colour of large spots for discrimination between artificial flowers, although the flies learned better to land on artificial flowers with a yellow spot colour than those with a blue spot colour. Since it is known that *E*. *tenax* flies prefer yellow, green and white flowers over blue and red flowers [39], it is uncertain whether the preference for large yellow over large blue spots is linked to the preference for overall flower colour or to that for pollen colour, the assumed target of the proboscis extension (see below).

Limitations in the spatial resolution of the visual system in *Eristalis* flies could lead to the failure of detection of the relevant stimuli in our experiments. The spots representing the target for the proboscis response were relatively small with a minimum diameter of 2mm. However, the minimal spot size to which *E*. *tenax* flies respond amounted to 0.2 mm and was largely independent of the background colour [40]. Few flies even extended their proboscis towards 0.1mm sized yellow spots which is about the size of one large pollen grain.

Some studies about the proboscis extension in *Eristalis* have been done using monochromatic light stimuli [12, 16], others using coloured cardboards [31–32, 37, 41] or coloured nutrients [4]; how these differences in stimuli affect the behaviour is unknown. The colour that triggers the innate proboscis reflex in *E*. *tenax* has originally and most exactly been tested using monochromatic light stimuli [12]. The essential studies of the proboscis extension reflex in *E*. *tenax* were done also with naïve individuals and showed that the imagoes extended the proboscis reflex towards monochromatic light stimuli only in the range between 510nm and 600nm wavelength [12]. The elicitation of the proboscis reflex was strongly inhibited, if only 10% of ultraviolet or blue monochromatic light was admixed to monochromatic yellow light to which 80% of the flies responded [12, 16].

Cevik and Erden [42] defined that the proboscis extension reflex (PER) is triggered when insects’ gustatory receptors contact appetitive stimuli. This definition suggests that all appetitive stimuli that are combined with an unconditioned reward might lead to a successful conditioning. Moreover, this definition ignores that honeybees, bumblebee as well as hoverflies are known to exhibit a proboscis reflex triggered by visual stimuli [10–12, 37]. The inability of visual colour conditioning in *Eristalis* flies seems to indicate that there is no need for learning in this context due to a very reliable pollen colour.

The results of this study provide evidence that the innate proboscis extension reflex towards yellow colours cannot be modified by absolute nor differential conditioning. If the natural stimulus triggering the proboscis reflex is constant, there is apparently no need for conditioning to other stimuli of the same modality. For example, the spontaneous colour choices of herbivorous butterflies are also fixed [43] which might be explained by the fact that the vast majority of green leaves are indeed green. Pollen has been thought to represent the natural target stimulus of the innate proboscis reflex in *E*. *tenax* [15], since most pollen grains of flowers pollinated by insects are yellow [44]. The *Eristalis* flies might benefit from the fixed innate proboscis reflex to yellow colour in terms of reliable finding pollen as an essential food source rich in protein [45]. Some flowering plants might benefit from exploiting the fixed preference and response to yellow colour in terms manipulating the flies’ movements on the flower or misdirecting them to nectar holders [31–32, 46]. Indeed, many nectar guides are yellow and UV-absorbing and thus mimic the predominant colour of pollen [17, 47]. Floral colour pattern displayed by pollen, anthers or floral guides are important features improving plant pollination [17, 47–51]. This study contributes thus another facet to the importance of floral guides to ensure plant reproduction through impact of pollinators’ behaviour. In this study only blue was tested as a conditioned stimulus. Similar experiments with differential conditioning including punishment with deep yellow and reward with red, green and light yellow colours revealed similar results (Sermon & Lunau, unpublished).

Flower visiting animals are well known for their learning capacities [52–53] enabling discrimination of rewarding and non-rewarding flowers and flower constant foraging behaviour [54–56]. Although Dukas [54] emphasized that the ecological context of learning is important for insects, he concluded that learning is probably a universal property of insects, which rely on learning for all major life functions. Limitations of learning in insects have been discussed in relation to intoxication with pesticides [36, 57–58], interference with innate preferences [59–60], training [61–62], and limitations by sensory capabilities [35, 63]. The results of this study indicate that the colour preference for yellow of the proboscis reflex is strong and cannot be modified by conditioning. *E*. *tenax* shows a preference for yellow colours also for the landing reaction, which, however can altered by training depending on the experimental conditions. The conditioning of the proboscis extension to monochromatic light stimuli was, however, successful in houseflies, *Musca domestica* [64] indicating that the absence of conditioning to colours in *E*. *tenax* is outstanding. The comparative study with seven genera of hoverflies demonstrate that the innate proboscis reflex towards yellow colours in *Eristalis* flies is exceptional even among flower-visiting hoverflies.

## Supporting information

S1 Fig Artificial flowers and experimental setups. Training and test artificial flowers of all experiments are shown against the background used.
(PDF)

S2 Fig Spectral reflectance properties of the tested colour stimuli and backgrounds. The spectral reflectance of colour stimuli and backgrounds is shown in the range of wavelength between 300nm and 700nm.
(TIF)

S1 Video Choice test of an *Eristalis tenax* fly trained to artificial flowers with a large-sized spot. The trained fly chooses between grey artificial flowers with a large spot of either blue or yellow colour following training to artificial flowers with large blue spot.
(WMV)
