# Supplementary material for: Limitations of learning in the proboscis reflex of the flower visiting syrphid fly Eristalis tenax
Source: PLoS One. 2018 Mar 20;13(3):e0194167. doi: 10.1371/journal.pone.0194167 (PMC5860702; doi:10.1371/journal.pone.0194167)

Dual choice of trained flies (proboscis reflex)

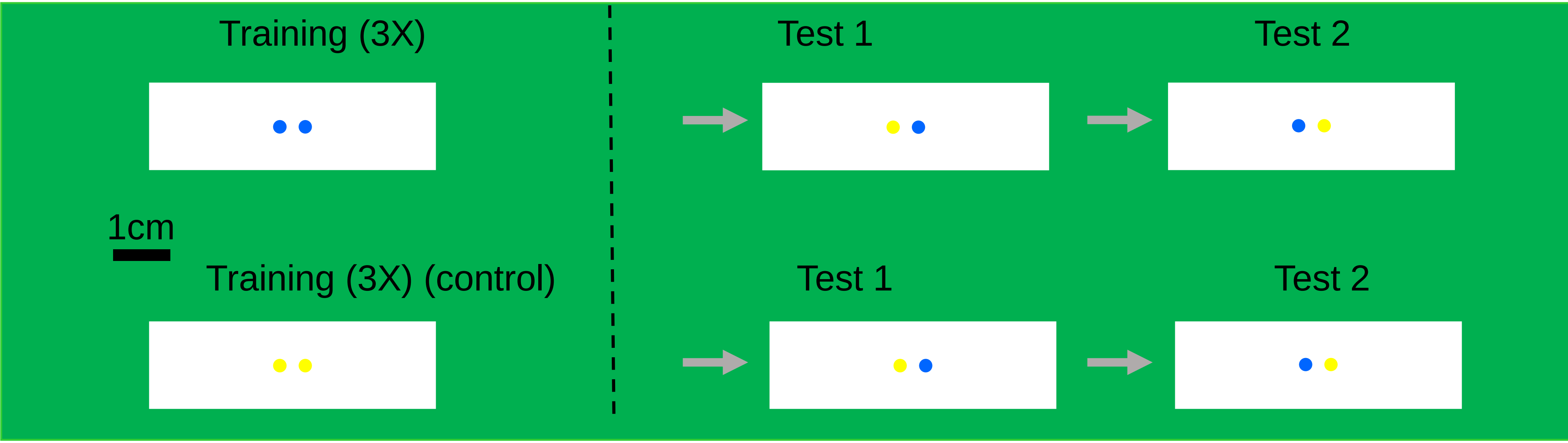

Dual choice of trained flies (direction of walking)

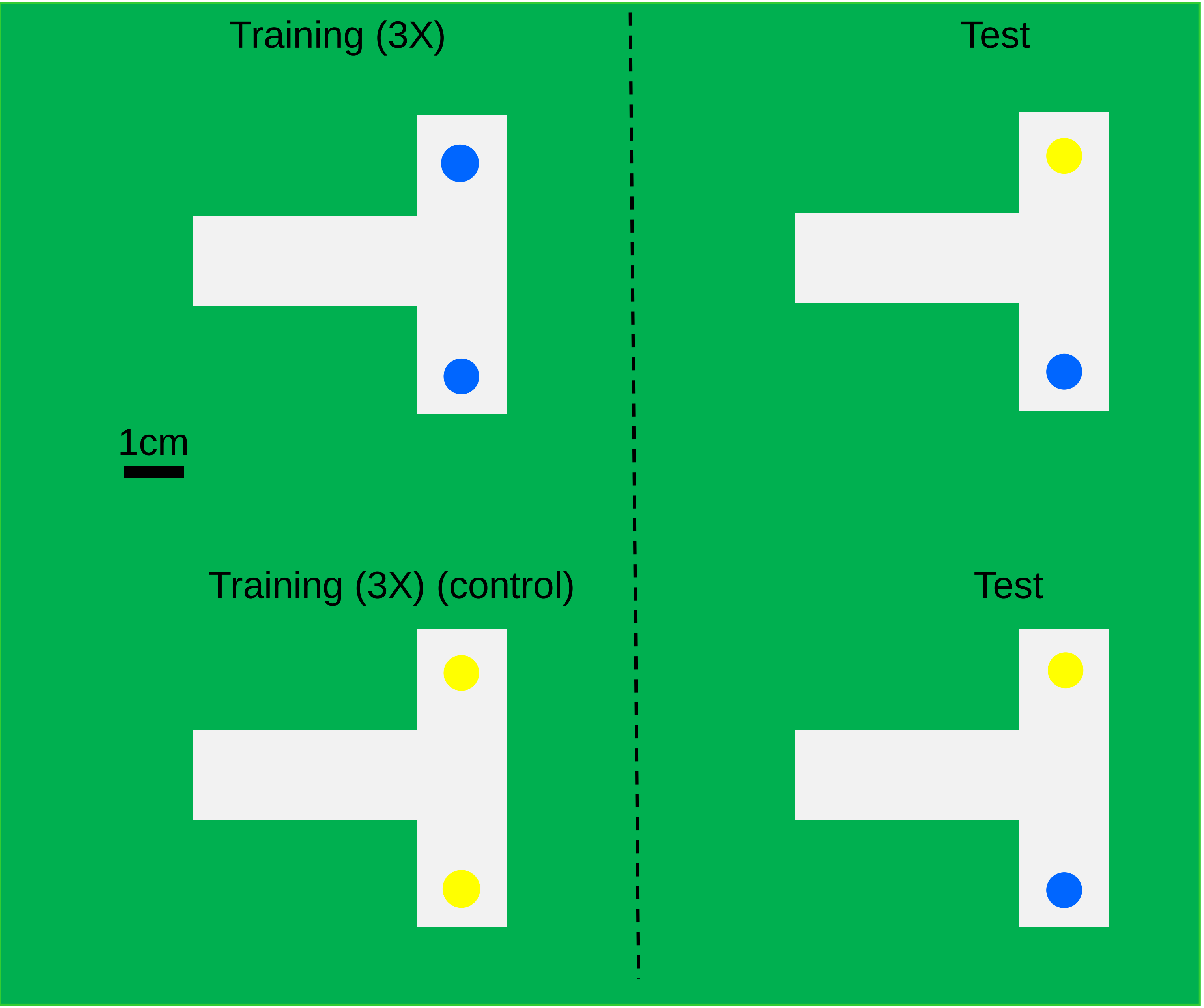

Absolute and differential conditioning

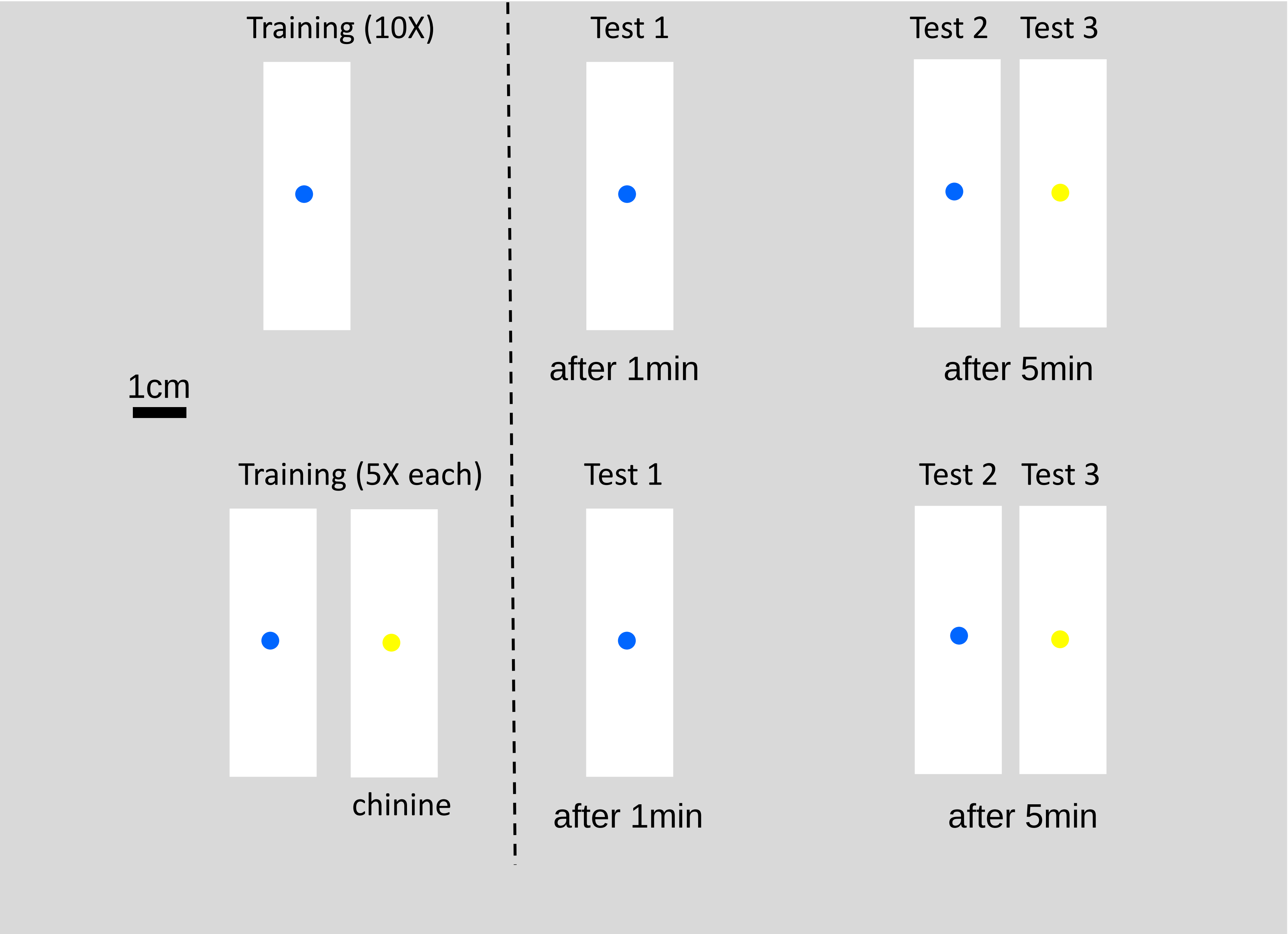

Proboscis extension reflex in experienced field-caught flies

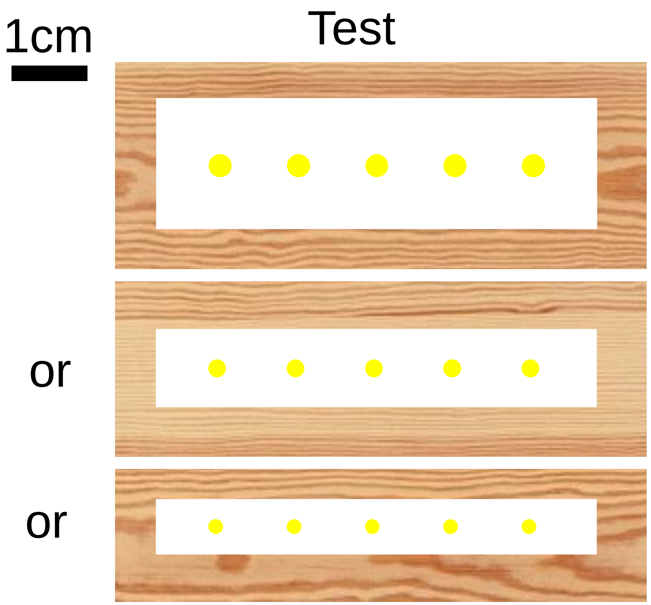

Landing response to artificial flowers with different spot colour of small spots

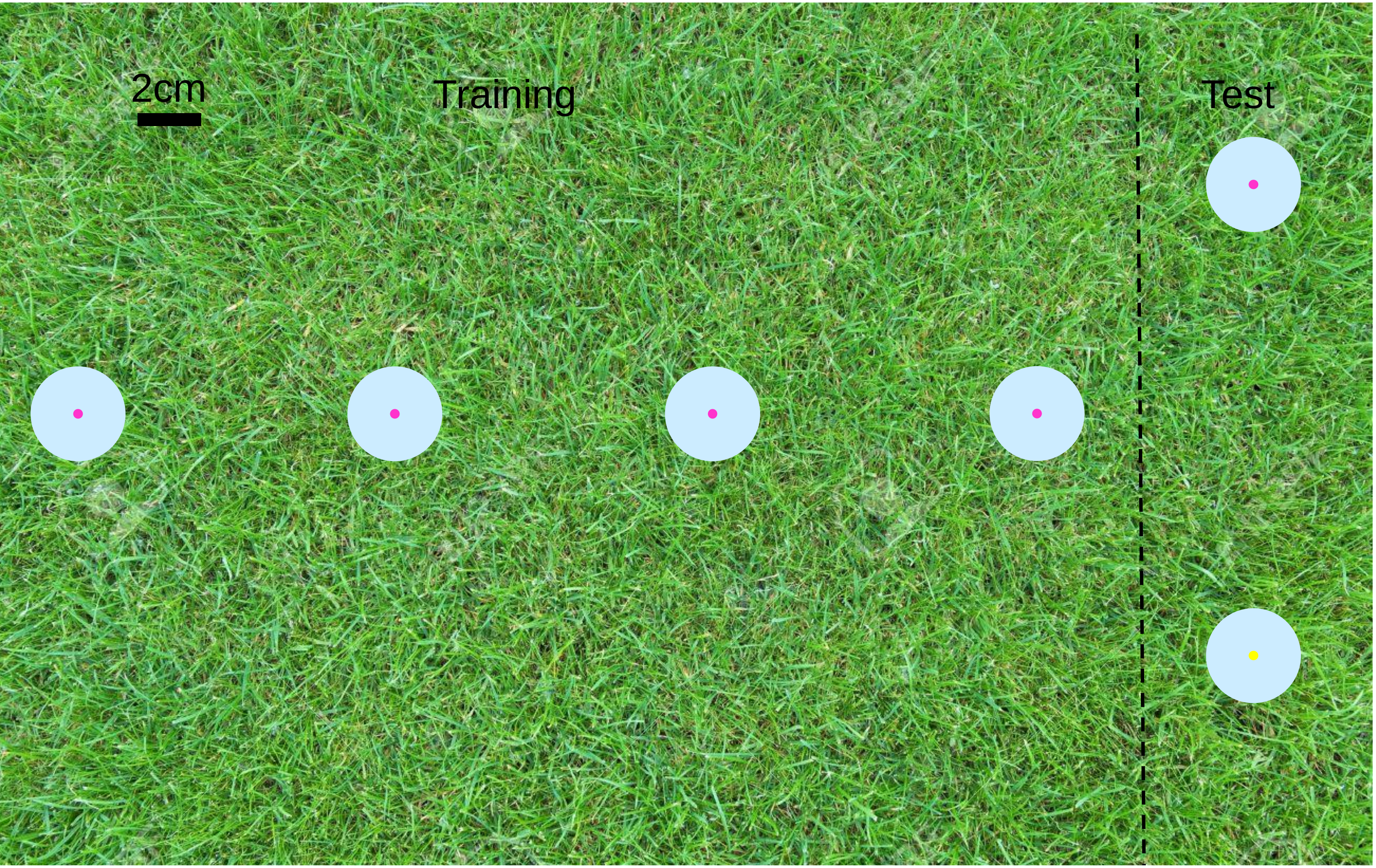

Landing response to artificial flowers with different spot colour of large spots

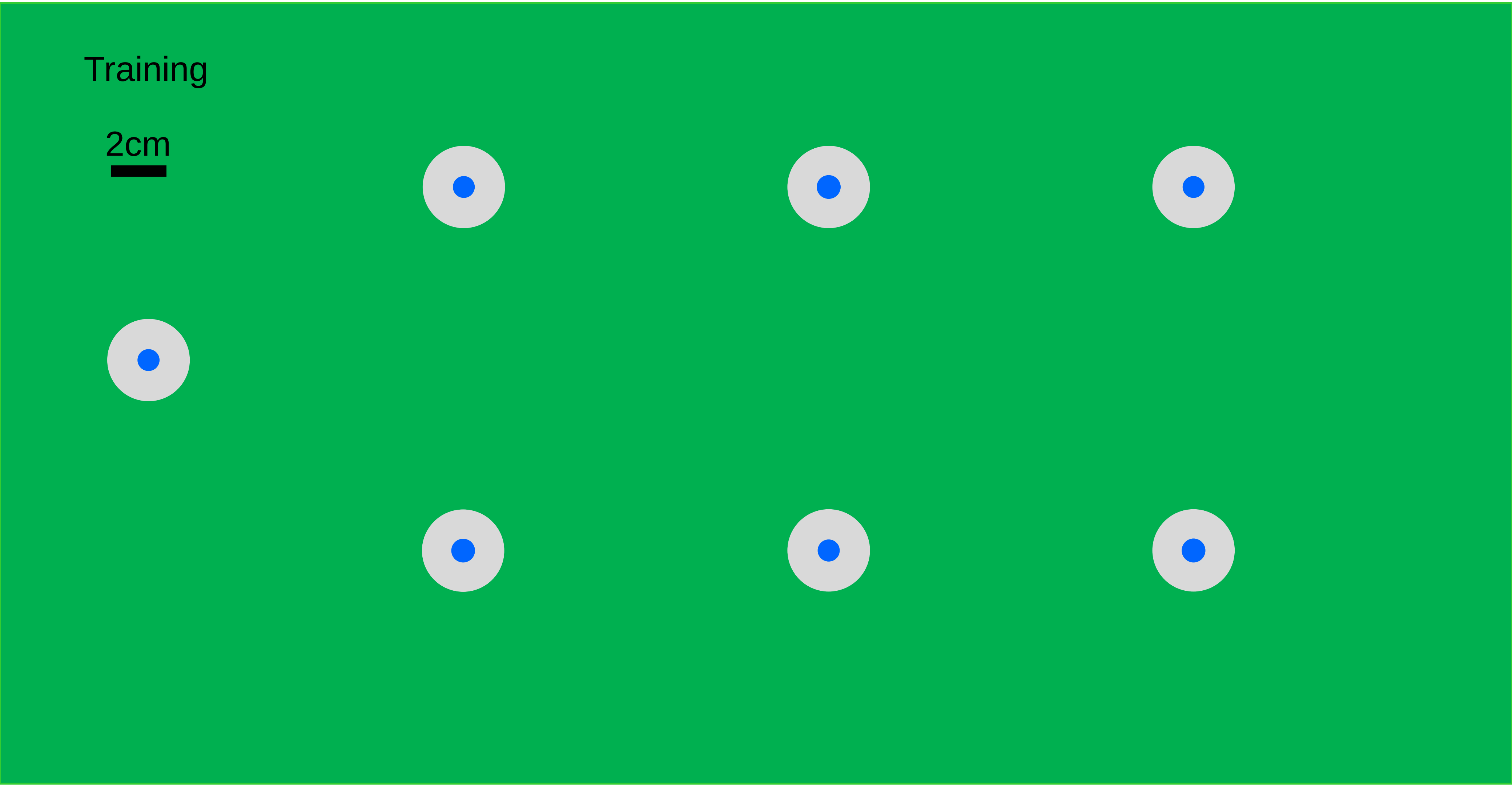

Landing response to artificial flowers with different spot colour of large spots

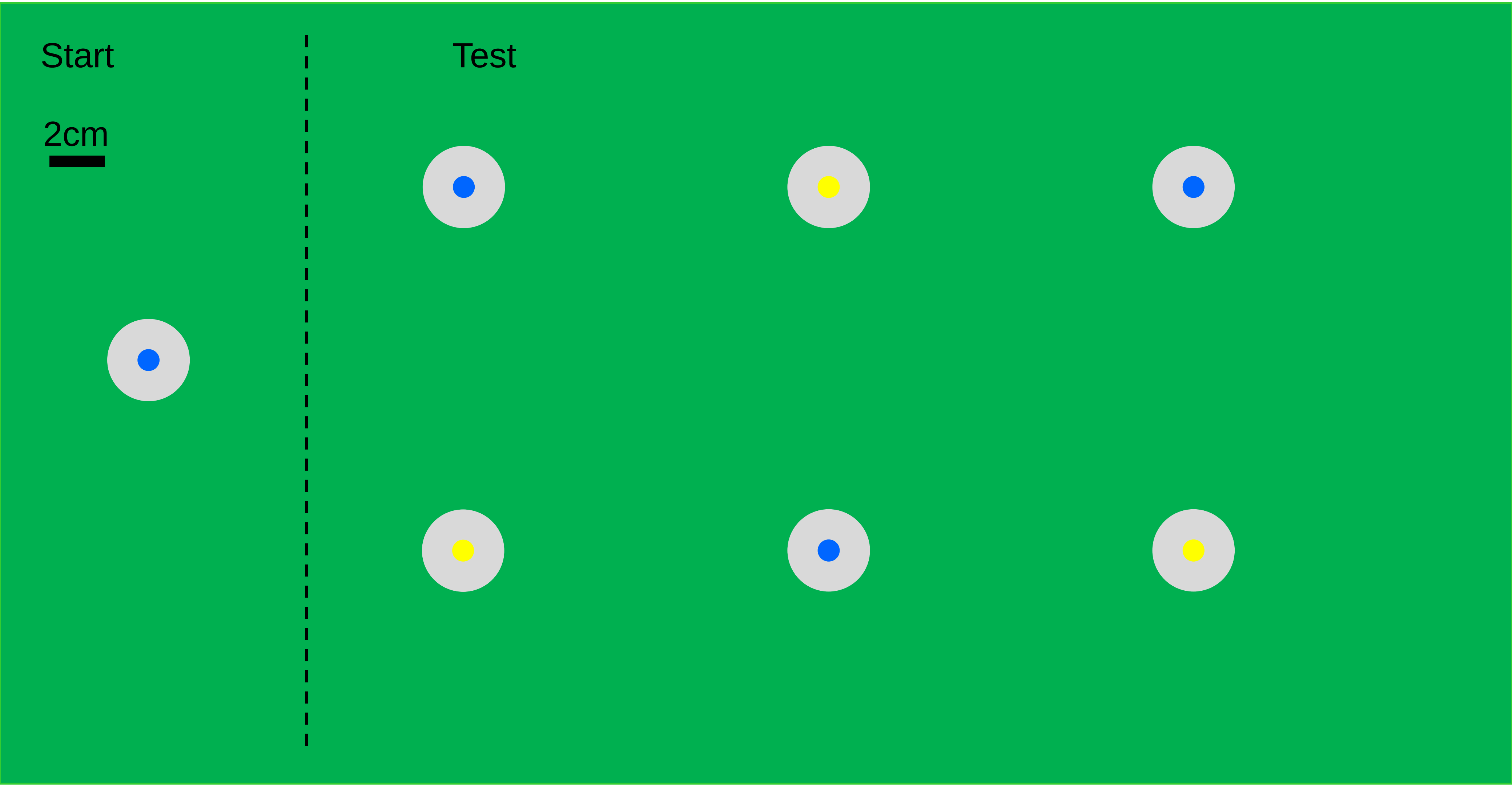

Supplement: S1 Fig — Training and test artificial flowers of all experiments are shown against the background used. (PDF) [file pone.0194167.s001.pdf]
