# Supplementary figures and images for: Limitations of learning in the proboscis reflex of the flower visiting syrphid fly Eristalis tenax
Source: PLoS One. 2018 Mar 20;13(3):e0194167. doi: 10.1371/journal.pone.0194167 (PMC5860702; doi:10.1371/journal.pone.0194167)

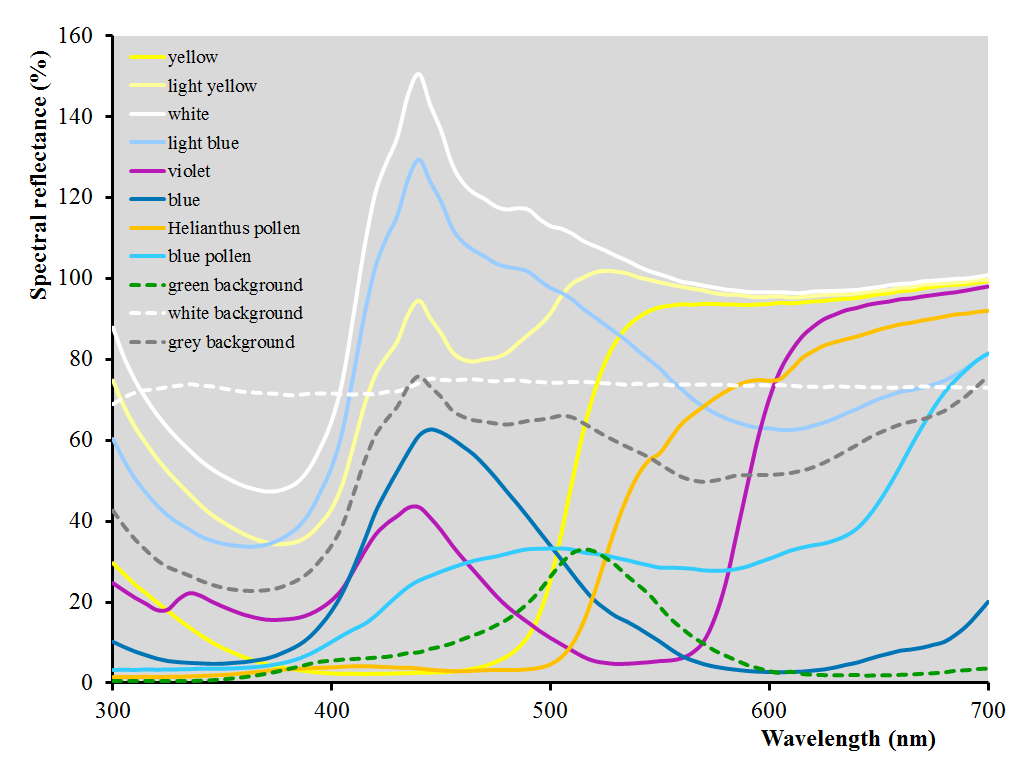

Supplement: S2 Fig — The spectral reflectance of colour stimuli and backgrounds is shown in the range of wavelength between 300nm and 700nm. (TIF) [file pone.0194167.s002.tif]
